# Supplementary material for: Effects of Polybrominated Diphenyl Ethers on Hormonal and Reproductive Health in E-Waste-Exposed Population: A Systematic Review
Source: Int J Environ Res Public Health. 2022 Jun 25;19(13):7820. doi: 10.3390/ijerph19137820 (PMC9265575; doi:10.3390/ijerph19137820)
Supplement: Supplementary file 1 [file ijerph-19-07820-s001.zip › Supplementary material S1 - Methods.pdf]

1: Search terms

Table S1 (a): Scopus

#1	Title, abstract and keywords	"polybrominated diphenyl*" OR "polybrominated biphenyl*" OR "diphenyl ether*" OR "biphenyl ether*" OR BDE* OR PBDE* OR halogen* OR organohalogen* OR POP OR POPS OR "persistent organic pollutant*" OR "Flame retard*" OR "fire retard*" OR fireproof* OR "fire proof*"
#2	Title, abstract and keywords	"E-waste" OR WEEE OR EEE OR "electronic waste" OR "waste electronic" OR "waste electric" OR "electronic recycling"
#3	Title, abstract and keywords	Health OR hormone* OR endocrine OR reproduct* OR development* OR damag* OR disorder* OR dysfunc* OR defic* OR impair* OR expos* OR effect* OR impact* OR influence* OR consequence* OR affect* OR condition* OR disrupt* OR associat* OR alter* OR factor*
#4		#1 AND #2 AND #3

Table S1 (b): Embase

#1	Title, abstract and keywords	"polybrominated diphenyl*" OR "polybrominated biphenyl*" OR "diphenyl ether*" OR "biphenyl ether*" OR BDE* OR PBDE* OR halogen* OR organohalogen* OR POP OR POPS OR "persistent organic pollutant*" OR "Flame retard*" OR "fire retard*" OR fireproof* OR "fire proof*"
#2	Title, abstract and keywords	"E-waste" OR WEEE OR EEE OR "electronic waste" OR "waste electronic" OR "waste electric" OR "electronic recycling"
#3	Title, abstract and keywords	Health OR hormone* OR endocrine OR reproduct* OR development* OR damag* OR disorder* OR dysfunc* OR defic* OR impair* OR expos* OR effect* OR impact* OR influence* OR consequence* OR affect* OR condition* OR disrupt* OR associat* OR alter* OR factor*
#4		#1 AND #2 AND #3

Table S1 (c): PubMed

#1	Title and abstract	"polybrominated diphenyl*" OR "polybrominated biphenyl*" OR "diphenyl ether*" OR "biphenyl ether*" OR BDE* OR PBDE* OR halogen* OR organohalogen* OR POP OR POPS OR "persistent organic pollutant*"*" OR "Flame retard*" OR "fire retard*" OR fireproof* OR "fire proof*"
	MeSH Terms	"Flame retardants" OR "Halogenated Diphenyl Ethers" OR "Persistent Organic Pollutants" OR "Polybrominated biphenyls"
#2	Title and abstract	"E-waste" OR WEEE OR EEE OR "electronic waste" OR "waste electronic" OR "waste electric" OR "electronic recycling"
	MeSH Terms	“Electronic waste"
#3	Title and abstract	Health OR hormone* OR endocrine OR reproduct* OR development* OR damag* OR disorder* OR dysfunc* OR defic* OR impair* OR expos* OR effect* OR

		impact* OR influence* OR consequence* OR affect* OR condition* OR disrupt* OR associat* OR alter* OR factor*
	MeSH Terms	Hormones OR "Infant health" OR "Reproductive health" OR "Sexual health"
#4		#1 AND #2 AND #3

2: The Navigation Guide Method for Grading Human Evidence

Figure S2 (a): Steps of the Navigation Guide

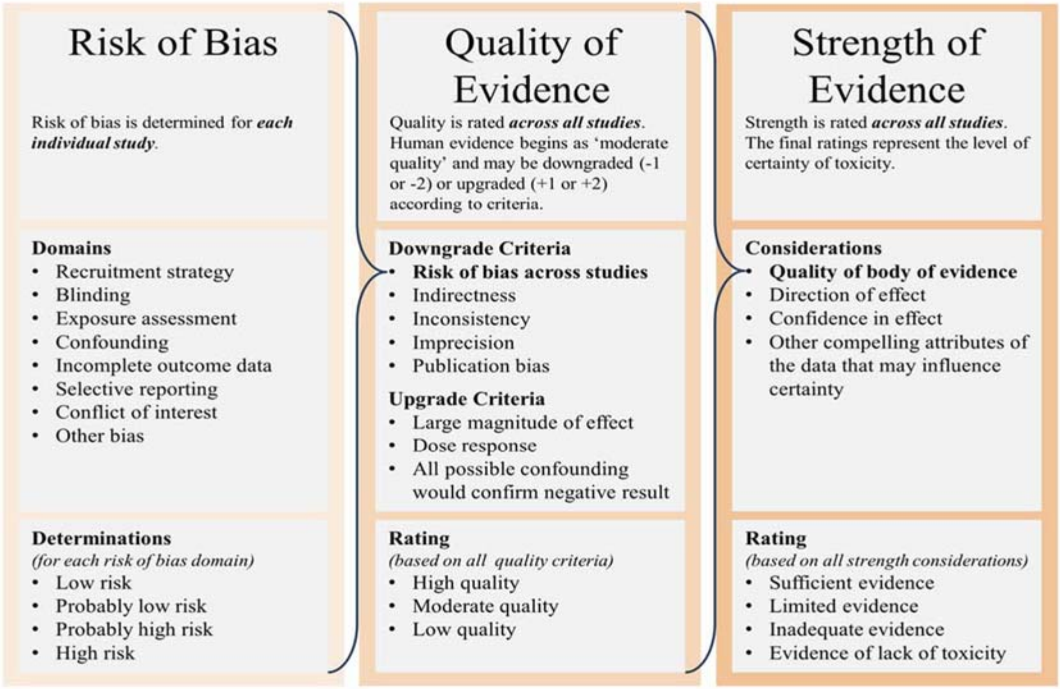

Table S2 (b): Rationale for final judgment of the Navigation Guide

Sufficient evidence of toxicity	The available evidence usually includes consistent results from well-designed, well-conducted studies, and the conclusion is unlikely to be strongly affected by the results of future studies. For human evidence a positive relationship is observed between exposure and outcome where chance, bias, and confounding, can be ruled out with reasonable confidence.
Limited Evidence of Toxicity	The available evidence is sufficient to determine the effects of the exposure, but confidence in the estimate is constrained by such factors as: the number, size, or quality of individual studies, the confidence in the effect, or inconsistency of findings across individual studies. As more information becomes available, the observed effect could change, and this change may be large enough to alter the conclusion. For human evidence a positive relationship is observed between exposure and outcome where chance, bias, and confounding cannot be ruled out with reasonable confidence.
Inadequate Evidence of Toxicity	Studies permit no conclusion about a toxic effect. The available evidence is insufficient to assess effects of the exposure. Evidence is insufficient because of: the limited number or size of studies, low quality of individual studies, or inconsistency of findings across individual studies. More information may allow an estimation of effects.
Evidence of Lack of Toxicity	The available evidence includes consistent results from well-designed, well-conducted studies, and the conclusion is unlikely to be strongly affected by the results of future studies. For human evidence more than one study showed no effect on the outcome of interest at the full range of exposure levels that humans are known to encounter, where bias and confounding can be ruled out with reasonable confidence. The conclusion is limited to the age at exposure and/or other conditions and levels of exposure studied.
